# Investigation of the association between interleukin-1β polymorphism and normal tension glaucoma

**Published:** 2007-05-14

**Authors:** Chun Yuan Wang, Ying-Cheng Shen, Chien-Hui Su, Fai-Yun Lo, Shi-Huang Lee, Hin-Yeung Tsai, Seng-Sheen Fan

**Affiliations:** 1 Department of Ophthalmology, Taichung Veterans General Hospital, National Yang-Ming University, Taichung, Taiwan, Republic of China; 2 Department of Life Science and Life Science Research Center, Tunghai University, Taichung, Taiwan, Republic of China; 3 Hung Kuang University, Taichung, Taiwan, Republic of China; 4 Overseas Chinese Institute of Technology, Taichung, Taiwan, Republic of China

## Abstract

**Purpose:**

In normal tension glaucoma (NTG), factors other than elevated intraocular pressure are likely to have a role in the pathogenesis of optic neuropathy. The potential similarities in cellular apoptosis leading to neurodegeneration between Alzheimer's disease and NTG were shown in recent studies. The interleukin-1β (IL-1β; -511) and IL-1β (+3953) polymorphisms were found to increase risk with Alzheimer's disease. The purpose of this study was to test the hypothesis that the IL-1β polymorphism is associated with NTG in the Chinese population.

**Methods:**

This is a cohort study in a Chinese population that involved 231 people with NTG and 245 healthy controls. Genomic DNA was amplified by a polymerase chain reaction, followed by the enzymatic restriction fragment length polymorphism technique. Patients and controls were genotyped for the C/T polymorphism at position -511 and +3953 of the IL-1β gene. Genotypes for NTG and control groups were compared for statistically significant differences.

**Results:**

There was no significant difference in genotype frequency or allele frequency distribution of the IL-1β gene polymorphisms (position -511 and +3953) between NTG patients and the control group (p >0.3).

**Conclusions:**

Our study showed no evidence for an association between the IL-1β (-511) and IL-1β (+3953) polymorphisms and NTG. The IL-1β gene polymorphisms (position -511 and +3953) may not play a key role in NTG pathogenesis in Chinese population.

## Introduction

Glaucoma is a degenerative optic neuropathy characterized by loss of retinal ganglion cells, cupping of the optic nerve head, and visual field defects often related to elevated intraocular pressure. The disease affects approximately 70 million people worldwide and is the second most common cause of blindness [1]. Factors other than IOP are likely to have a role in the pathogenesis of glaucomatous optic neuropathy, particularly in individuals with normal tension glaucoma (NTG). NTG is a subtype of primary open angle glaucoma (POAG) and accounts for one-third of all cases of POAG [2]. Patients with NTG show IOP measurements within the statistically normal range, and these patients usually present late in life after a visual field defect has occurred. A genetic approach is needed to identify those at risk of developing NTG.

Recent laboratory evidence shows a connection between abnormal autoimmunity and NTG, suggesting NTG may be a glaucomatous condition affected by antibodies damaging retinal tissue and inducing apoptosis. Wax et al. [3] found deposition of IgG, IgA, and retinal antibodies in the retinal ganglion cell of NTG patients. Fellman et al. [4] noted that patients with NTG and rheumatoid disease have a high level of serum antibodies to rhodopsin and heat shock protein 60.

Shinji et al. [5] found interleukin-1β (IL-1β) plays an important role in mediating ischemic and excitotoxic damage in the retina in glaucoma. Proinflammatory cytokines, such as IL-1, as well as other indicators of microglial activation, have been suggested as drivers of neuropathological changes in several neurodegenerative conditions.

Vickers et al. [6] presented evidence that the neuronal pathology of Alzheimer's disease contributes to an aberrant regenerative response of nerve cells triggered by the gradual compression and physical damage to axons within beta-amyloid plaques that form in the brain. Glaucoma may be a chronic neurodegeneration like Alzheimer's disease, because similar evidence also indicates there is beta-amyloid build-up in retinal ganglion cells in rats with experimental glaucoma [7,8]. In this regard, glaucoma may be viewed as a chronic neurodegenerative disease similar to Alzheimer's disease, and a slow build up of beta-amyloid in the ganglion cell may eventually trigger cell death and optic nerve axon loss. There is evidence that the IL-1 protein may act to promote the development of beta-amyloid deposits [9-12].

Researchers have noted several polymorphic regions in the IL-1β gene. A C/T polymorphism at position -511 of the IL-1β gene in the promoter region has been reported to be associated with Alzheimer's disease, with the IL-1β(-511) T allele polymorphism found to increase the risk for late-onset Alzheimer's disease [13-15]. The other polymorphism in IL-1β (at position +3953 in exon 5) has shown an association with the risk for Alzheimer's disease [16].

NTG may be a chronic neurodegeneration like Alzheimer's disease. Given the potential similarities in cellular events leading to neurodegeneration between Alzheimer's disease and glaucoma, we hypothesized that the IL-1β (-511) and IL-1β (+3953) polymorphisms, because of the effect on IL-1 protein expression, may predispose affected individuals to glaucoma. We therefore sought to investigate the distribution of IL-1β (-511) and IL-1β (+3953) polymorphisms in NTG patients and compare them with a healthy control population.

## Methods

### Subjects

Subjects were recruited at the outpatient clinic in the Department of Ophthalmology at the Veterans General Hospital, Taichung, Taiwan from January 2004 to February 2007. NTG patients were approached as they visited the clinic for previously scheduled visits and were enrolled after consenting to participate in the study. Normal control subjects were recruited during their visits to the outpatient clinic for various other reasons. Written informed consent was obtained from all study subjects prior to enrollment. The study was carried out with the approval of the Human Study Committee of the Veterans General Hospital.

All participants received comprehensive ophthalmologic examinations including visual acuity testing with refraction, IOP measurement, Humphrey 30-2, slit lamp examination, and dilated slit lamp stereobiomicroscopy. Comprehensive ophthalmologic history and longitudinal follow data were also obtained for each individual. The definition for NTG included the presence of typical glaucomatous optic neuropathy with compatible visual field defects (arcuate, Bjerrum, Seidel and/or paracentral scotoma and/or nasal step on Humphrey 30-2), open anterior chamber angle, and absence of any contributing ocular or systemic disorders. Patients with NTG had untreated IOP measurements that were consistently 21 mmHg or lower on diurnal test and at follow-up.

Unrelated control subjects were recruited from clinic patients who were seeking treatment for senile cataract, floater, refractive errors, or itchy eye. All normal control subjects had no systemic disease and no family history of glaucoma. They were excluded from glaucoma using the same criteria of diagnosis as the NTG patients after the same ophthalmic examination procedure.

### DNA preparation and genotype identification

Blood samples were collected from each subject (5 ml) and genomic DNA was isolated using the Qiagen QiaAmp Blood mini kit (Qiagen, Valencia, CA). IL-1β C(-511)T and C(+3953)T genotyping of genomic DNA were determined with polymerase chain reaction-restriction fragment length polymorphism (PCR-RFLP) technique. A 304 bp PCR fragment of the IL-1β (-511) in the promoter region was amplified using the following primers: F5'-TGG CAT TGA TCT GGT TCA TC-3' and R5'-GTT TAG GAA TCT TCC CAC TT-3'. PCR conditions were as follows: a denaturing step of 95 °C for 10 min, then 35 cycles of 95 °C for 45 s, 60 °C for 45 s, 72 °C for 1 min, and a final incubation at 72 °C for 5 min. The products were digested with *Bsu*36I (New England Biolabs, Inc., Beverly, MA) at 37 °C for 3 h and were run on ethidium bromide-stained 2% agarose gel. This gave products that either remained intact (C allele) or were cut into two fragments of 190 and 114 bp (T allele).

The polymorphic region containing the *Taq*I (New England Biolabs, Inc.) restriction site at position +3953 within exon 5 of the IL-1β gene was amplified using the following primers: F5'-GTT GTC ATC AGA CTT TGA CC-3' and 5'-TTC AGT TCA TAT GGA CCA GA-3'. The PCR conditions were the same as described in the previous. The products were digested with *Taq*I at 65 °C for 3 h. *Taq*I digestions of the 249 bp fragments were cut into two fragment of 135 and 114 bp (allele C) or remained intact (allele T).

### Statistical analysis

Genotype and allele frequencies between the control and NTG groups were compared using the chi-square test and Fisher's exact test, respectively. Age and gender were compared between the control and NTG groups using the Student's t-test and Fisher's exact test, respectively. Odds ratios were computed to assess the strength of association between the presence of each genotype and the clinical diagnosis of NTG. A p value of less than 0.05 was defined to be of statistical significance. All statistical analyses were performed using SPSS 10.0 (SPSS Inc., Chicago, IL).The power calculation was carried out according to the method by Schlesselman [17].

## Results

The study consisted of 231 NTG patients (127 men, 104 women) and 245 normal controls (135 men, 110 women). The mean age was 70 years for the NTG patients (range 31-85) and 71 years for the controls (range 30-85). There was no difference between the control and NTG groups in age (p>0.05, t-test) and gender (p>0.05, Fisher's exact test). The mean and standard deviation of the maximum IOP was 17.5±2.1 mmHg for the NTG patients and 16.9 ±2.3 mmHg for the control subjects. No deviations from Hardy-Weinberg equilibrium could be seen in NTG patients and control studies.

The genotype and allele frequencies of the IL-1β (-511) and IL-1β (+3953) polymorphism in NTG and control subjects are presented in Table 1. There was no statistically significant difference in genotype frequency (GF) or allele frequency (AF) distribution of the two polymorphisms between NTG and control subjects. (IL-1β -511 AF: p=0.3, GF: p=0.4; IL-1β +3953 AF: p=0.88, GF: p=0.98)

**Table 1:** Genotype and allele frequencies of interleukin-1β (-511) and interleukin-1β (+3953).

Genotype	NTG (%) n= 231	Control (%) n=245	χ^2^	p-value
IL-1β (-511)				
Genotype				
C/C	70 (30.3%)	61 (24.9%)		
C/T	108 (46.8%)	125 (51.0%)	1.77	0.4
T/T	53 (22.9%)	59 (24.1%)		
Allele				
C	248 (53.7%)	247 (50.4%)	1.02	0.3
T	214 (46.3%)	243 (49.6%)		
IL-1β (+3953)				
Genotype				
C/C	214 (93.8%)	226 (92.2%)		
C/T	16 ( 6.9%)	18 ( 7.3%)	0.03	0.98
T/T	1 ( 0.4%)	1 ( 0.4%)		
Allele				
C	444 (96.1%)	470 (95.9%)	0.02	0.88
T	18 ( 3.9%)	20 ( 4.1%)		

There was no statistically significant difference in genotype frequency (GF) or allele frequency (AF) distribution of the two polymorphisms between NTG and control subjects. The breakdown was as follows: For IL-1β -511, the AF was p=0.3 and the GF was p=0.4; for IL-1? +3953, the AF was p=0.88 and the GF was p=0.98.

The statistical power over 80% suggested that the probability of detecting a difference could be believable in this sample size.

## Discussion

Mutations in three genes (myocilin, optineurin, and WDR36) have been implicated in NTG [18-22]. Mutation in the optineurin gene was initially reported in 16.7% of families with hereditary primary open angle glaucoma (POAG), with most of them having NTG [19]. Aung et al. [23] and Powell et al. [24] reported that NTG demonstrates an association with polymorphisms of the OPA1 gene on chromosome 3, which is responsible for dominant optic atrophy in the Caucasian population [25]. Many more important gene variants have recently been associated with glaucoma risk, such as apolipoprotein E (*APOE*) [26,27], endothelin receptor type A gene (*EDNRA*) [28,29], IL-1α [30], methylenetetrahydrofolate reductase (*MTHFR*) [31,32], and beta-adrenergic receptors [33]. However, these genes cannot interpret the overall inheritance susceptibility of NTG pathogenesis. The other associations involved in the development of NTG should be further investigated.

Recent evidence indicates a close link between chronic neurodegenerative disease and IL-1β expression. Polymorphism in the IL-1 gene clusters have been shown in myasthenia gravis, multiple sclerosis, Parkinson's disease, temporal lobe epilepsy with hippocampal sclerosis and Alzheimer's disease [34-38]. NTG may be a chronic neurodegeneration like Alzheimer's disease, considering the potential similarities in cellular events leading to neurodegeneration between Alzheimer's disease and glaucoma. There is evidence that IL-1β upregulates the production and processing of β-amyloid protein in neurons [39] as well as the expression and activity of acetylcholine esterase [40]. Furthermore, it has been shown that IL-1β activated microglia selectively killed cholinergic neurons in Alzheimer's patients [41]. The death of retinal ganglion cells in glaucoma involving chronic β-amyloid neurotoxicity mimics Alzheimer's disease at the molecular level [7]. Our study was designed to see whether patients with NTG had a higher risk of developing the disease as a result of their IL-1β genotype.

NTG has been considered a subtype of POAG, sharing many similar characteristics. However, some studies show a connection between abnormal autoimmunity and NTG, suggesting the disease is affected more by antibodies damaging retinal ganglion cells and inducing apoptosis [42]. Lin et al. [43] found that IL-1β (+3953) T allele was significantly more common in POAG patients than in control subjects in a Chinese population. However, there was no association between the IL-1β (position -511 and +3953) polymorphisms and NTG in our study. The dissimilar findings revealed that the pathogenesis and effect of IL-1β may be different between NTG and POAG. IL-1β may not have a significant role in the pathogenesis of the optic neuropathy.

Genes coding for the two isoforms of IL-1 (IL-1α and IL-1β) and for the IL-1 receptor antagonist (IL-1RA) are located within the IL1 gene cluster at chromosomal locus 2q13 [44]. Polymorphisms within this gene cluster have been associated with a large variety of human diseases [45]. Some of these polymorphisms have been shown to alter the amount of IL-1 produced [46-49]. These allelic variants are known to alter function. The C/T polymorphism at position -511 in the promoter region of IL-1β regulates the production of IL-1β protein and in vitro synthetic capacity of C/C genotype carriers are lower than that of C/T of T/T carriers [50]. The homozygosity for the IL-1β (+3953T) allele has been associated with a fourfold increase in the production of IL-1β when compared to homozygosity for IL-1β (+3953C) allele [47].

Lack of association between IL-1β genetic polymorphism and NTG was observed in our study of a Chinese population. In our control group, the allele frequencies of the IL-1β (-511) were similar to those previous reported in other controls [43,51,52]. The frequency of IL-1β (-511) C allele and IL-1β (+3953) T allele were significantly lower among Chinese populations compared to Caucasians [53]. The observed negative association could be due to the low frequency of the IL-1β (-511) C allele and IL-1β (+3953) T allele in a Chinese population. Further studies with NTG cohort of different ethnic background are required to further define this association. We cannot exclude the possibility of IL-1β (-511) and IL-1β (+3953) polymorphism being associated with NTG in different ethnic population or the likelihood of an association between NTG and another IL-1β polymorphism. The lack of an association of the common polymorphisms of the IL-1β with NTG in Chinese population suggests this factor may not have a significant role in the pathogenesis of the optic neuropathy.

In conclusion, we found no significant associations between polymorphisms in the IL-1β (-511 and +3953) and NTG in a Chinese population. However, the possibility of other mutations or sequence change in the IL-1β gene cannot be excluded. Further genetic studies of NTG are necessary to investigate the development of the neurodegenerative process.

## References

[1] Quigley HA Broman AT The number of people with glaucoma worldwide in 2010 and 2020. Br J Ophthalmol 2006 90 262 7 16488940 10.1136/bjo.2005.081224 PMC1856963

[2] Kamal D Hitchings R Normal tension glaucoma--a practical approach. Br J Ophthalmol 1998 82 835 40 9924383 10.1136/bjo.82.7.835 PMC1722650

[3] Wax MB Tezel G Edward PD Clinical and ocular histopathological findings in a patient with normal-pressure glaucoma. Arch Ophthalmol 1998 116 993 1001 9715678 10.1001/archopht.116.8.993

[4] Fellman RL Tezel G Wax MB Effects of methotrexate treatment on serum immunoreactivity of a patient with normal-pressure glaucoma. Am J Ophthalmol 1999 127 724 5 10372887 10.1016/s0002-9394(99)00020-3

[5] Yoneda S Tanihara H Kido N Honda Y Goto W Hara H Miyawaki N Interleukin-1beta mediates ischemic injury in the rat retina. Exp Eye Res 2001 73 661 7 11747366 10.1006/exer.2001.1072

[6] Vickers JC Dickson TC Adlard PA Saunders HL King CE McCormack G The cause of neuronal degeneration in Alzheimer's disease. Prog Neurobiol 2000 60 139 65 10639052 10.1016/s0301-0082(99)00023-4

[7] McKinnon SJ Glaucoma: ocular Alzheimer's disease? Front Biosci 2003 8 s1140 56 12957857 10.2741/1172

[8] McDowell TL Symons JA Ploski R Forre O Duff GW A genetic association between juvenile rheumatoid arthritis and a novel interleukin-1 alpha polymorphism. Arthritis Rheum 1995 38 221 8 7848312 10.1002/art.1780380210

[9] Wisniewski T Frangione B Apolipoprotein E: a pathological chaperone protein in patients with cerebral and systemic amyloid. Neurosci Lett 1992 135 235 8 1625800 10.1016/0304-3940(92)90444-c

[10] Rebeck GW Reiter JS Strickland DK Hyman BT Apolipoprotein E in sporadic Alzheimer's disease: allelic variation and receptor interactions. Neuron 1993 11 575 80 8398148 10.1016/0896-6273(93)90070-8

[11] Schmechel DE Saunders AM Strittmatter WJ Crain BJ Hulette CM Joo SH Pericak-Vance MA Goldgaber D Roses AD Increased amyloid beta-peptide deposition in cerebral cortex as a consequence of apolipoprotein E genotype in late-onset Alzheimer disease. Proc Natl Acad Sci USA 1993 90 9649 53 8415756 10.1073/pnas.90.20.9649 PMC47627

[12] Ma J Yee A Brewer HB Jr Das S Potter H Amyloid-associated proteins alpha 1-antichymotrypsin and apolipoprotein E promote assembly of Alzheimer beta-protein into filaments. Nature 1994 372 92 4 7969426 10.1038/372092a0

[13] Du Y Dodel RC Eastwood BJ Bales KR Gao F Lohmuller F Muller U Kurz A Zimmer R Evans RM Hake A Gasser T Oertel WH Griffin WS Paul SM Farlow MR Association of an interleukin 1 alpha polymorphism with Alzheimer's disease. Neurology 2000 55 480 3 10953177 10.1212/wnl.55.4.480

[14] Grimaldi LM Casadei VM Ferri C Veglia F Licastro F Annoni G Biunno I De Bellis G Sorbi S Mariani C Canal N Griffin WS Franceschi M Association of early-onset Alzheimer's disease with an interleukin-1alpha gene polymorphism. Ann Neurol 2000 47 361 5 10716256

[15] Nicoll JA Mrak RE Graham DI Stewart J Wilcock G MacGowan S Esiri MM Murray LS Dewar D Love S Moss T Griffin WS Association of interleukin-1 gene polymorphisms with Alzheimer's disease. Ann Neurol 2000 47 365 8 10716257 PMC3833599

[16] Hedley R Hallmayer J Groth DM Brooks WS Gandy SE Martins RN Association of interleukin-1 polymorphisms with Alzheimer's disease in Australia. Ann Neurol 2002 51 795 7 12112093 10.1002/ana.10196

[17] Schlesselman JJ. Case-control studies. New York: Oxford University Press; 1982.

[18] Stone EM Fingert JH Alward WL Nguyen TD Polansky JR Sunden SL Nishimura D Clark AF Nystuen A Nichols BE Mackey DA Ritch R Kalenak JW Craven ER Sheffield VC Identification of a gene that causes primary open angle glaucoma. Science 1997 275 668 70 9005853 10.1126/science.275.5300.668

[19] Rezaie T Child A Hitchings R Brice G Miller L Coca-Prados M Heon E Krupin T Ritch R Kreutzer D Crick RP Sarfarazi M Adult-onset primary open-angle glaucoma caused by mutations in optineurin. Science 2002 295 1077 9 11834836 10.1126/science.1066901

[20] Monemi S Spaeth G DaSilva A Popinchalk S Ilitchev E Liebmann J Ritch R Heon E Crick RP Child A Sarfarazi M Identification of a novel adult-onset primary open-angle glaucoma (POAG) gene on 5q22.1. Hum Mol Genet 2005 14 725 33 15677485 10.1093/hmg/ddi068

[21] Weisschuh N Neumann D Wolf C Wissinger B Gramer E Prevalence of myocilin and optineurin sequence variants in German normal tension glaucoma patients. Mol Vis 2005 11 284 7 http://www.molvis.org/molvis/v11/a33/15851979

[22] Sripriya S Nirmaladevi J George R Hemamalini A Baskaran M Prema R Ve Ramesh S Karthiyayini T Amali J Job S Vijaya L Kumaramanickavel G OPTN gene: profile of patients with glaucoma from India. Mol Vis 2006 12 816 20 http://www.molvis.org/molvis/v12/a92/16885925

[23] Aung T Ocaka L Ebenezer ND Morris AG Brice G Child AH Hitchings RA Lehmann OJ Bhattacharya SS Investigating the association between OPA1 polymorphisms and glaucoma: comparison between normal tension and high tension primary open angle glaucoma. Hum Genet 2002 110 513 4 12073024 10.1007/s00439-002-0711-9

[24] Powell BL Toomes C Scott S Yeung A Marchbank NJ Spry PG Lumb R Inglehearn CF Churchill AJ Polymorphisms in OPA1 are associated with normal tension glaucoma. Mol Vis 2003 9 460 4 http://www.molvis.org/molvis/v9/a58/14551537

[25] Yao W Jiao X Hejtmancik JF Leske MC Hennis A Nemesure B Barbados Family Study Group. Evaluation of the association between OPA1 polymorphisms and primary open-angle glaucoma in Barbados families. Mol Vis 2006 12 649 54 http://www.molvis.org/molvis/v12/a73/16785854

[26] Vickers JC Craig JE Stankovich J McCormack GH West AK Dickinson JL McCartney PJ Coote MA Healey DL Mackey DA The apolipoprotein epsilon4 gene is associated with elevated risk of normal tension glaucoma. Mol Vis 2002 8 389 93 http://www.molvis.org/molvis/v8/a46/12379839

[27] Mabuchi F Tang S Ando D Yamakita M Wang J Kashiwagi K Yamagata Z Iijima H Tsukahara S The apolipoprotein E gene polymorphism is associated with open angle glaucoma in the Japanese population. Mol Vis 2005 11 609 12 http://www.molvis.org/molvis/v11/a72/16110302

[28] Ishikawa K Funayama T Ohtake Y Kimura I Ideta H Nakamoto K Yasuda N Fukuchi T Fujimaki T Murakami A Asaoka R Hotta Y Kanamoto T Tanihara H Miyaki K Mashima Y Association between glaucoma and gene polymorphism of endothelin type A receptor. Mol Vis 2005 11 431 7 http://www.molvis.org/molvis/v11/a50/15988412

[29] Kim SH Kim JY Kim DM Ko HS Kim SY Yoo T Hwang SS Park SS Investigations on the association between normal tension glaucoma and single nucleotide polymorphisms of the endothelin-1 and endothelin receptor genes. Mol Vis 2006 12 1016 21 http://www.molvis.org/molvis/v12/a114/16971893

[30] Wang CY Shen YC Lo FY Su CH Lee SH Lin KH Tsai HY Kuo NW Fan SS Polymorphism in the IL-1alpha (-889) locus associated with elevated risk of primary open angle glaucoma. Mol Vis 2006 12 1380 5 http://www.molvis.org/molvis/v12/a155/17149369

[31] Junemann AG von Ahsen N Reulbach U Roedl J Bonsch D Kornhuber J Kruse FE Bleich S C677T variant in the methylentetrahydrofolate reductase gene is a genetic risk factor for primary open-angle glaucoma. Am J Ophthalmol 2005 139 721 3 15808177 10.1016/j.ajo.2004.09.081

[32] Mabuchi F Tang S Kashiwagi K Yamagata Z Iijima H Tsukahara S Methylenetetrahydrofolate reductase gene polymorphisms c.677C/T and c.1298A/C are not associated with open angle glaucoma. Mol Vis 2006 12 735 9 http://www.molvis.org/molvis/v12/a82/16862068

[33] Inagaki Y Mashima Y Fuse N Funayama T Ohtake Y Yasuda N Murakami A Hotta Y Fukuchi T Tsubota K Polymorphism of beta-adrenergic receptors and susceptibility to open-angle glaucoma. Mol Vis 2006 12 673 80 http://www.molvis.org/molvis/v12/a75/16785856

[34] Griffin WS Mrak RE Interleukin-1 in the genesis and progression of and risk for development of neuronal degeneration in Alzheimer's disease. J Leukoc Biol 2002 72 233 8 12149413 PMC3835694

[35] Huang D Pirskanen R Hjelmstrom P Lefvert AK Polymorphisms in IL-1beta and IL-1 receptor antagonist genes are associated with myasthenia gravis. J Neuroimmunol 1998 81 76 81 9521608 10.1016/s0165-5728(97)00161-6

[36] Kanemoto K Kawasaki J Miyamoto T Obayashi H Nishimura M Interleukin (IL)1beta, IL-1alpha, and IL-1 receptor antagonist gene polymorphisms in patients with temporal lobe epilepsy. Ann Neurol 2000 47 571 4 10805326

[37] Muller N Ackenheil M Psychoneuroimmunology and the cytokine action in the CNS: implications for psychiatric disorders. Prog Neuropsychopharmacol Biol Psychiatry 1998 22 1 33 9533165 10.1016/s0278-5846(97)00179-6

[38] Sciacca FL Ferri C Vandenbroeck K Veglia F Gobbi C Martinelli F Franciotta D Zaffaroni M Marrosu M Martino G Martinelli V Comi G Canal N Grimaldi LM Relevance of interleukin 1 receptor antagonist intron 2 polymorphism in Italian MS patients. Neurology 1999 52 1896 8 10371542 10.1212/wnl.52.9.1896

[39] Sheng JG Ito K Skinner RD Mrak RE Rovnaghi CR Van Eldik LJ Griffin WS In vivo and in vitro evidence supporting a role for the inflammatory cytokine interleukin-1 as a driving force in Alzheimer pathogenesis. Neurobiol Aging 1996 17 761 6 8892349 10.1016/0197-4580(96)00104-2 PMC3886636

[40] Li Y Liu L Kang J Sheng JG Barger SW Mrak RE Griffin WS Neuronal-glial interactions mediated by interleukin-1 enhance neuronal acetylcholinesterase activity and mRNA expression. J Neurosci 2000 20 149 55 10627591 10.1523/JNEUROSCI.20-01-00149.2000 PMC6774108

[41] McMillian M Kong LY Sawin SM Wilson B Das K Hudson P Hong JS Bing G Selective killing of cholinergic neurons by microglial activation in basal forebrain mixed neuronal/glial cultures. Biochem Biophys Res Commun 1995 215 572 7 7487994 10.1006/bbrc.1995.2503

[42] Gutteridge IF Normal tension glaucoma: diagnostic features and comparisons with primary open angle glaucoma. Clin Exp Optom 2000 83 161 72 12472448 10.1111/j.1444-0938.2000.tb04910.x

[43] Lin HJ Tsai SC Tsai FJ Chen WC Tsai JJ Hsu CD Association of interleukin 1beta and receptor antagonist gene polymorphisms with primary open-angle glaucoma. Ophthalmologica 2003 217 358 64 12913327 10.1159/000071352

[44] Nicklin MJ Barton JL Nguyen M FitzGerald MG Duff GW Kornman K A sequence-based map of the nine genes of the human interleukin-1 cluster. Genomics 2002 79 718 25 11991722 10.1006/geno.2002.6751

[45] Haukim N Bidwell JL Smith AJ Keen LJ Gallagher G Kimberly R Huizinga T McDermott MF Oksenberg J McNicholl J Pociot F Hardt C D'Alfonso S Cytokine gene polymorphism in human disease: on-line databases, supplement 2. Genes Immun 2002 3 313 30 12209358 10.1038/sj.gene.6363881

[46] Pociot F Molvig J Wogensen L Worsaae H Nerup JA TaqI polymorphism in the human interleukin-1 beta (IL-1 beta) gene correlates with IL-1 beta secretion in vitro. Eur J Clin Invest 1992 22 396 402 1353022 10.1111/j.1365-2362.1992.tb01480.x

[47] Hall SK Perregaux DG Gabel CA Woodworth T Durham LK Huizinga TW Breedveld FC Seymour AB Correlation of polymorphic variation in the promoter region of the interleukin-1 beta gene with secretion of interleukin-1 beta protein. Arthritis Rheum 2004 50 1976 83 15188375 10.1002/art.20310

[48] Maury CP Liljestrom M Laiho K Tiitinen S Kaarela K Hurme M Anaemia of chronic disease in AA amyloidosis is associated with allele 2 of the interleukin-1beta-511 promoter gene and raised levels of interleukin-1beta and interleukin-18. J Intern Med 2004 256 145 52 15257727 10.1111/j.1365-2796.2004.01353.x

[49] Dominici R Cattaneo M Malferrari G Archi D Mariani C Grimaldi LM Biunno I Cloning and functional analysis of the allelic polymorphism in the transcription regulatory region of interleukin-1 alpha. Immunogenetics 2002 54 82 6 12037600 10.1007/s00251-002-0445-9

[50] Santtila S Savinainen K Hurme M Presence of the IL-1RA allele 2 (IL1RN*2) is associated with enhanced IL-1beta production in vitro. Scand J Immunol 1998 47 195 8 9519856 10.1046/j.1365-3083.1998.00300.x

[51] Hurme M Santtila S IL-1 receptor antagonist (IL-1Ra) plasma levels are co-ordinately regulated by both IL-1Ra and IL-1beta genes. Eur J Immunol 1998 28 2598 602 9710237 10.1002/(SICI)1521-4141(199808)28:08<2598::AID-IMMU2598>3.0.CO;2-K

[52] Tarlow JK Blakemore AI Lennard A Solari R Hughes HN Steinkasserer A Duff GW Polymorphism in human IL-1 receptor antagonist gene intron 2 is caused by variable numbers of an 86-bp tandem repeat. Hum Genet 1993 91 403 4 8500797 10.1007/BF00217368

[53] Tseng LH Chen PJ Lin MT Shau WY Chaung SM Martin PJ Hansen JA Single nucleotide polymorphisms in intron 2 of the human interleukin-1 receptor antagonist (IL-1Ra) gene: further definition of the IL-1 beta and IL-1Ra polymorphisms in North American Caucasians and Taiwanese Chinese. Tissue Antigens 2001 57 318 24 11380940 10.1034/j.1399-0039.2001.057004318.x

